# Symbiont coordinates stem cell proliferation, apoptosis, and morphogenesis of gut symbiotic organ in the stinkbug-*Caballeronia* symbiosis

**DOI:** 10.3389/fphys.2022.1071987

**Published:** 2023-01-04

**Authors:** Seonghan Jang, Yu Matsuura, Kota Ishigami, Peter Mergaert, Yoshitomo Kikuchi

**Affiliations:** ^1^ Bioproduction Research Institute, National Institute of Advanced Industrial Science and Technology, Hokkaido Center, Sapporo, Japan; ^2^ Division of Life Sciences, Korea Polar Research Institute, Incheon, South Korea; ^3^ Tropical Biosphere Research Center, University of the Ryukyus, Nishihara, Okinawa, Japan; ^4^ Graduate School of Agriculture, Hokkaido University, Sapporo, Japan; ^5^ Université Paris-Saclay, CEA, CNRS, Institute for Integrative Biology of the Cell (I2BC), Gif-sur-Yvette, France

**Keywords:** apoptosis, cell cycle, *Caballeronia*, bean bug, symbiosis, morphogenesis, intestinal stem cell

## Abstract

The bean bug *Riptortus pedestris* obtains a specific bacterial symbiont, *Caballeronia insecticola* (*Burkholderia insecticola*), from the environmental soil and harbors it in the posterior midgut region that is composed of hundreds of crypts. While newly hatched aposymbiotic insects possess primordial midgut crypts with little or no lumen, colonization of *C. insecticola* triggers swift development of the symbiotic organ, forming enlarged and opened crypts, and the symbiont subsequently fills the luminal cavities of those mature crypts. The cellular processes of crypt development triggered by *C. insecticola* colonization are poorly understood. Here we identified a fundamental mechanism of the symbiont-mediated midgut development by investigating cell cycles of intestinal epithelial cells. Intestinal stem cells of the bean bug are located and proliferate at the crypt base. Differentiated enterocytes migrate upward along the epithelial cell layer of the crypt as the midgut develops, induction of apoptosis in enterocytes primarily occurred on the tip side of the crypts, and apoptotic cells then eventually were shed from the crypts into the hemolymph. The proliferation rate of the stem cells at the base of the crypts was low while a high apoptotic rate was observed at the crypt tip in aposymbiotic insects, resulting in undeveloped short crypts. On the contrary, the gut-colonizing *C. insecticola* promoted the proliferation of the stem cells at the base of crypts and simultaneously inhibited apoptosis at the tip of crypts, resulting in a net growth of the crypts and the generation of a crypt lumen that becomes colonized by the bacterial symbiont. These results demonstrated that the *Caballeronia* symbiont colonization induces the development of the midgut crypts *via* finely regulating the enterocyte cell cycles, enabling it to stably and abundantly colonize the generated spacious crypts of the bean bug host.

## Introduction

The development of morphological phenotypes of animals and plants can be drastically altered by biotic and/or abiotic factors. One of the driving forces causing morphological innovations and developmental plasticity in eukaryotes is a symbiotic association with microorganisms (Gilbert, 2016). Microorganisms inhabit virtually every environment and many are intimately associated with animal and plant hosts. While some of them cause serious diseases as pathogens, a growing number of reports suggest that many microbes are beneficial for their hosts. In many cases, these microbial partners influence host development primarily through nutritional interactions (Douglas, 1998; Vance, 2001). These animals and plants acquire symbiotic bacteria from their parents or the environment and symbiont infection stimulates the development of specific tissues dedicated to the maintenance of symbionts, the so-called symbiotic organs (Fronk and Sachs, 2022). For instance, nitrogen-fixing rhizobium symbionts infect leguminous plants *via* the root hairs, inducing a cascade of processes leading to root nodule development, where symbionts colonize and exchange nutrients with the host (Poole et al., 2018). A well-studied example of an animal-microbe symbiotic system is the squid-*Vibrio* bioluminescent symbiosis. In the Hawaiian bobtail squid *Euprymna scolopes*, cell wall components of symbiotic bacteria promote apoptosis of host cells in the luminescent organ, leading to dramatic morphological alterations and maturation of the symbiotic organ harboring only the light-emitting strains of *Vibrio fischeri* (Visick et al., 2000; Koropatnick et al., 2004; Troll et al., 2009; Essock-Burns et al., 2021).

Gut symbiosis is one of the most common forms of symbioses in most animal taxa (Dillon and Dillon, 2004; Li et al., 2008; Egerton et al., 2018). Recent studies in model animals such as mice, zebrafish, and fruit flies, have revealed that gut symbiotic bacteria contribute to diverse aspects of host metabolism and development, including the morphogenesis of the intestinal tract (Nayak, 2010), the development and homeostasis of immunity (Wu and Wu, 2012), and even behavioral changes *via* the brain-gut microbial axis (Liberti and Engel, 2020; Kim et al., 2021). In mice, gut microbes stimulate the morphogenesis of intestinal villi by promoting the vascular network as well as cell proliferation (Stappenbeck et al., 2002; Nigro and Sansonetti, 2015). In *Drosophila melanogaster*, bacterial infection induces the proliferation and differentiation of intestinal stem cells (ISCs) *via* the Janus kinase-signal transducer and activator of transcription (JAK-STAT) pathway, contributing to gut homeostasis (Buchon et al., 2009; Jiang et al., 2009; Bonfini et al., 2016).

Most animals, including the above model systems, acquire and maintain dozens or sometimes hundreds of microbial species inside their gut (Moran et al., 2019). On the other hand, examples of mono-specific gut symbioses have been described in stinkbugs (Heteroptera: Pentatomomorpha). Most phytophagous species of stinkbugs develop numerous sac-like tissues, called crypts, at the posterior midgut region, wherein only a single bacterial species is maintained (Kikuchi et al., 2008). Some stinkbugs are tightly associated with bacterial symbionts belonging to the Gammaproteobacteria and have achieved strong host-symbiont specificity by evolving sophisticated vertical transmission mechanisms from mother to offspring (Fukatsu and Hosokawa, 2002; Kaiwa et al., 2014; Hosokawa et al., 2016). In contrast, the other stinkbugs accommodate specifically *Caballeronia* (*Burkholderia*) species of the Betaproteobacteria, which are horizontally acquired from the environment by every new offspring generation, involving rigorous partner-choice mechanisms (Kikuchi et al., 2011a; Takeshita and Kikuchi, 2017). Previous studies in the bean bug *Riptortus pedestris* (Fabricius, 1775) (Pentatomomorpha: Coreoidea: Alydidae), a model system for the stinkbug-microbe symbiosis without vertical transmission, have revealed that the host-symbiont specificity is achieved by at least two mechanisms. First, the entrance to the crypt-bearing symbiotic gut region is extremely narrow and filled with a mucus-like matrix, preventing the passage of food materials (Ohbayashi et al., 2015). This narrow gate, named the constricted region, sorts out the preferred symbiont from environmental microbiota that are ingested during feeding. Interestingly, the constricted region is completely closed after symbiont colonization in the midgut crypts to prevent secondary infections (Kikuchi et al., 2020). Second, some non-symbiotic bacteria can pass through the constricted region, but efficient microbe-microbe competition eliminates them within the luminal region of the crypts, and only a specific group of symbiotic bacteria (i.e., *Caballeronia* symbiont species) is able to stably colonize the symbiotic organ (Itoh et al., 2019).

Aposymbiotic (symbiont-free) hatchlings of the bean bug have undeveloped midgut crypts with little or no lumen, but infection by the *Caballeronia insecticola* (Takeshita et al., 2018) (Pseudomonadota: Betaproteobacteria: Burkholderiaceae) in the symbiotic organ triggers a rapid morphogenic response, leading to the formation of enlarged sac-like crypts, and the resulting spacious luminal cavities of the mature crypts are then colonized by the symbiont (Kikuchi et al., 2007; Kikuchi and Fukatsu, 2014). Such an enlarged midgut organ in symbiont-infected insects has been reported also in many other stinkbug species (Kikuchi et al., 2009; Tada et al., 2011; Oishi et al., 2019). Nevertheless, the detailed cellular processes of crypt development and symbiotic morphology stimulated by the symbiotic bacteria are poorly characterized. Focusing on the *Riptortus-Caballeronia* model system, we therefore studied here how epithelial cells in the crypts respond to the symbiont infection and how crypt morphogenesis proceeds. We visualized cell division, cell cycle, and apoptosis *via* microscopy observation and quantification of molecular markers in the midgut crypts in symbiotic and aposymbiotic insects. We demonstrated that symbiont colonization activates stem cell proliferation of the crypts and also prolongs cellular life span of developed epithelial cells by suppressing apoptosis, thereby establishing the drastic morphological changes of the crypts that enable the stable symbiotic association.

## Materials and methods

### Insect rearing and symbiont infection

The bean bug *R. pedestris* was reared in the laboratory under a long-day regimen (16 h light and 8 h dark) at 25°C in Petri dishes (90 mm diameter and 20 mm height). Soybean seeds and distilled water containing 0.05% ascorbic acid (DWA) were supplied to the bugs and replaced with new ones every 2 days. To establish *Caballeronia*-harboring symbiotic insects, *in vitro* cultured *C. insecticola* cells were diluted in DWA (10^7^ cells/ml), cotton pads were soaked with the bacterial suspension and provided to freshly molted second instar nymphs. To observe the gut development or the host’s cell cycle, green fluorescence protein (GFP)-labelled *C. insecticola* strain RPE225 or non-fluorescent wild-type *C. insecticola* strain RPE75 were administrated to the bean bugs, respectively (Kikuchi et al., 2011b; Kikuchi and Fukatsu, 2014).

### Observation of gut morphogenesis

Morphologies of the symbiotic organ of aposymbiotic and symbiotic bean bugs were observed under a laser scanning microscope (TCS SP8; Leica). The symbiotic insects were infected by *C. insecticola* strain RPE225 cells to visualize symbiont localization *in vivo*. The aposymbiotic insects were maintained in a symbiont-free state by feeding soybean and sterilized DWA. The symbiotic organs of insects 12, 24, 48, and 72 h after *C. insecticola* symbiont infection (*n* = 10 for each time point) were dissected in phosphate-buffered saline (PBS, pH 7.4) using fine forceps under a stereomicroscope (S8APO; Leica Microsystems). The isolated organs were then transferred into 1.5 ml microcentrifuge tubes, fixed with 4% paraformaldehyde solution in PBS (4% PFA-PBS) for 15 min, and incubated for 30 min with 1 μg/ml 4′6-diamidino-2-phenylindole (DAPI) (Thermo Fisher Scientific) and Alexa Fluor™ 647 phalloidin (Thermo Fisher Scientific) to stain the insect’s nucleus and cytoskeleton, respectively. The midgut samples were then gently placed on a glass-bottom dish (Matsunami), immersed in ProLong Gold Antifade Mountant (Thermo Fisher Scientific), and covered with a coverglass for observation. Symbiotic organs were observed under the confocal microscope using a ×40 magnification oil objective (×40/1.3 HC PL Apo CS oil).

### Quantitative PCR

To estimate the number of midgut cells indirectly, the copy number of a housekeeping gene, elongation factor 1 alpha (*Ef1ɑ*), was measured by quantitative PCR (qPCR). DNA was extracted from the dissected symbiotic organs of aposymbiotic and symbiotic insects (*n* = 18 for each), and qPCR was performed using primers, 5′-CCT GCA TCC GTT GCT TTT GT-3′ and 5′-GGC ATC GAG GGC TTC AAT AA-3′ (Kim et al., 2013), the KAPA SYBR FAST qPCR Master Mix Kit (KAPA Biosystems), and the Roche LightCycler 96 System (Roche). The PCR temperature profile was 40 cycles of 95°C for 10 s, 60°C for 15 s, and 72°C for 15 s. The gene copy number was calculated based on a standard curve for the *Ef1ɑ* gene containing 10, 10^2^, 10^3^, 10^4^, 10^5^, 10^6^, and 10^7^ copies per reaction of the target PCR product.

### Measurement of numerical parameters of midgut crypts

The symbiotic organs of aposymbiotic and symbiotic insects (*n* = 10 for each) were dissected in PBS under the stereomicroscope and whole symbiotic organs were photographed by a digital camera connected to the microscope (S8APO equipped with EZ3; Leica Microsystems). The total length and width of symbiotic organs were measured using the line selection tool of ImageJ software (Schneider et al., 2012; Rueden et al., 2017) and the number of midgut crypts were manually counted by placing dots on all crypts. To measure the area of midgut crypts, microscopic images of partially enlarged midgut from three insect individuals, stained with DAPI and phalloidin as above, were taken by the confocal microscope (TCS SP8; Leica Microsystems), and the total area of randomly selected crypts (*n* = 9) was measured using the area selection tool of ImageJ software (Supplementary Figure S1).

### Quantification of ISCs

The ISCs of the symbiotic organ were visualized by staining with the thymidine analogue EdU, which is incorporated into newly synthesized DNA of replicating cells, using the Click-iT™ EdU imaging kit with Alexa Fluor™ 594 (Thermo Fisher Scientific). For this analysis, the aposymbiotic and symbiotic bean bugs at the second and third instar nymphal stages were dissected in PBS and the symbiotic organs were isolated from the abdomen (*n* = 3 for each). The isolated midgut samples were transferred into 1.5 ml microcentrifuge tubes and incubated with 20 μM solution of EdU in Grace’s insect medium (Thermo Fisher Scientific) for 1 h. The symbiotic organs were washed three times with PBS and fixed in 4% PFA-PBS for 15 min. The fixed midguts were washed three times by PBS and permeabilized for 10 min with 0.5% Triton X-100 in PBS (PBST). To detect EdU positive cells (ISCs), the permeabilized midgut samples were incubated with Click-iT^Ⓡ^ reaction cocktail (100 μl Click-iT reaction buffer, 800 μl CuSO_4_, and 100 μl 1X Click-iT^Ⓡ^ reaction buffer additive) for 30 min under dark conditions. The midgut samples were then incubated with Hoechst 33342 for 10 min to counterstain nuclei of the host midgut cells. The stained midguts were transferred into the glass-bottom dish and observed under the confocal microscope. The number of ISCs was determined by counting EdU^+^ cells in a whole symbiotic organ or each midgut crypt. To measure the number of ISCs in a crypt, images were taken by enlarging the part of the crypts, and the number of EdU^+^ cells was counted from 9 randomly selected crypts.

### Quantification of mitotic cells

The mitotic cells of the symbiotic organ were visualized by immunostaining of phospho-histone 3 (PH3) with an anti-phospho histone H3 (Ser10) polyclonal (anti-PH3) antibody (Cell Signaling Technology). The symbiotic organ was dissected from aposymbiotic and symbiotic insects at the second or third instar (*n* = 3 for each), transferred into 1.5 ml microcentrifuge tubes, fixed in 4% PFA-PBS for 15 min, and permeabilized with PBST for 30 min. The midgut samples were then blocked with a blocking solution [1% bovine serum albumin (BSA) in PBST] for 30 min and incubated with 1/2000 diluted anti-PH3 antibody in the blocking solution for 1 h at RT. Unbound primary antibodies were washed with PBS and the midgut samples were further incubated with 1/500 diluted Goat Anti-rabbit IgG (H&L) Alexa Fluor™ 488 (Thermo Fisher Scientific) in the blocking solution for 1 h at room temperature under dark conditions. The midguts were washed three times with PBS and incubated with Hoechst 33342 (Thermo Fisher Scientific) for 10 min. The prepared samples were placed on a glass-bottom dish and PH3-derived signals were observed under the confocal microscope using a ×40 magnification oil objective (×40/1.3 HC PL Apo CS oil). The number of mitotic cells was determined by counting PH3^+^ cells in 9 randomly selected midgut crypts.

### Quantification of apoptotic cells

Cell apoptosis in the symbiotic organ was visualized by the TdT-mediated dUTP-biotin nick end labeling (TUNEL) assay using the Click-iT™ Plus TUNEL Assay kit (Thermo Fisher Scientific). The dissected symbiotic organs of aposymbiotic and symbiotic insects (*n* = 3 for each) were fixed in 4% PFA-PBS for 15 min and washed three times with PBS. The fixed midgut samples were permeabilized by PBST for 15 min and washed with deionized water. The permeabilized organs were then incubated in TdT reaction buffer for 10 min at 37°C. After incubation, TdT reaction buffer was removed and the midgut samples were treated with TdT reaction mixture for 1 h at 37°C, then washed three times, and immediately incubated with Click-iT™ Plus TUNEL reaction cocktail for 30 min at 37°C under dark conditions. Then the midguts were washed three times with PBS and incubated with Hoechst 33342 (Thermo Fisher Scientific) for 10 min to stain host nuclei. The fluorescence signal of apoptotic cells was observed under the confocal microscope using a ×40 magnification oil objective (×40/1.3 HC PL Apo CS oil) lens. The number of apoptotic cells was determined by counting fluorescence-labeled cells in 9 randomly selected midgut crypts.

### Transmission electron microscopy

The symbiotic organs of aposymbiotic and symbiotic bean bugs at the third instar nymphal stage were carefully dissected in a fixative solution (0.1 M sodium phosphate buffer containing 2.5% glutaraldehyde, pH 7.4). The isolated midguts were pre-fixed in the fixative solution at 4°C overnight and post-fixed in 2% osmium tetroxide at 4°C for 1 h. The fixed midgut samples were serially dehydrated with ethanol and then embedded in Epon812 resin (TAAB). Ultrathin sections were obtained by an ultramicrotome (EM UC7; Leica Microsystems), mounted on a copper mesh, stained with uranyl acetate and lead citrate, and observed under a transmission electron microscope (H-7600; Hitachi).

### Statistical analysis

To analyze the statistical difference between aposymbiotic and symbiotic bean bugs, the Mann-Whitney *U* test was applied to the crypt size, crypt number, *Ef1ɑ* gene copy number, midgut length, midgut width, and nuclei number. All statistical analyses were performed using the program Prism 9 (https://www.graphpad.com).

## Results

### *Caballeronia* symbiont induces development of the symbiotic organ

**FIGURE 1:**
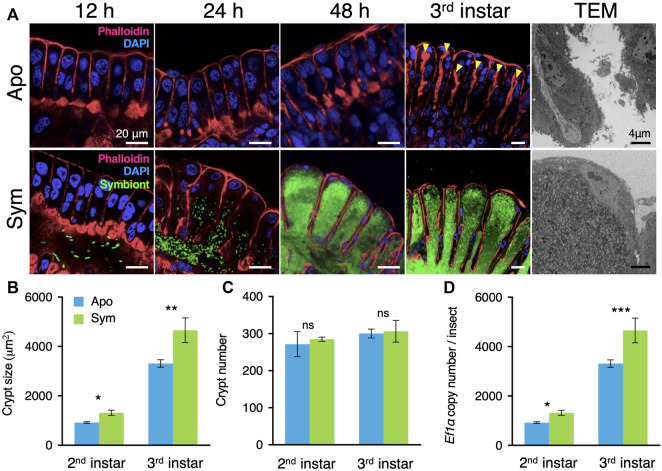
*Caballeronia* symbionts induce crypt development. **(A)** Comparison of crypt development between aposymbiotic and symbiotic insects. In aposymbiotic insects, the crypt lumen is not developed even 48 h after molting to the second instar and becomes visible in the third instar (arrowheads). By contrast, the crypt lumen is well developed within 24 h after symbiont inoculation, and crypts become enlarged with symbiont proliferation inside their lumen. Blue, host nucleus; red, cytoskeleton; green, GFP-labelled symbionts. TEM images of aposymbiotic and symbiotic insects are shown on the right side. **(B)** The total area of the midgut crypts (*n* = 10). **(C)** The number of crypts per insect (*n* = 10). **(D)** Determination by qPCR of the copy number of the host’s elongation factor 1α (ef1α) gene per symbiotic organ, per insect (*n* = 18). Mean ± SD is shown. Data was statistically analyzed by the Mann-Whitney *U* test (ns, non-significant; *, *p* < 0.05; **, *p* < 0.01; ***, *p* < 0.001). An example image of the crypt size measurement is shown in Supplementary Figure S1. Apo, aposymbiotic; Sym, symbiotic insects.

The bean bug acquires the *Caballeronia* symbiont at the second instar nymph stage from the environmental soil. The symbiotic midgut organ of the second instar nymphs before acquiring symbiotic bacteria was primordial, having a transparent, short, and thin morphology (Supplementary Figures S1, S2). The gut morphology of symbiont-free aposymbiotic insects remained undeveloped during insect growth until the third instar nymph (Figure 1A). However, when the bean bugs obtained the *Caballeronia* symbiont, the symbiotic organ initiated a drastic morphological differentiation. The colonization in the symbiotic organ by *Caballeronia* symbiont rapidly proceeded after oral administration of the bacteria. The GFP-labelled symbiont cells immediately passed the midgut regions M1 to M4B and entered the symbiotic organ M4 within 24 h (Figure 1A). The *Caballeronia* then expeditiously proliferated in the M4 and occupied most space of the midgut crypts in 48 h after infection (Figure 1A). The whole symbiotic organ was then fully colonized by *Caballeronia* cells at the third instar nymph stage (Figure 1A). The morphological development of the symbiotic organ was synchronous with this rapid symbiont colonization. Already after 24 h of infection, the aspect of the symbiotic organ changed and turned partially opaque (Supplementary Figure S2). Within 48 h of symbiont infection, the color of the symbiotic organ became remarkably yellowish and the total length and width of the M4 symbiotic organ became longer and wider compared to the organ in aposymbiotic insects (Supplementary Figures S2, S3). At the third instar nymph stage, the symbiotic organ developed further in symbiotic insects, becoming significantly longer and wider than those of aposymbiotic insects (Supplementary Figures S3A, S3B). These results apparently demonstrate that the morphological development of the symbiotic organ in the bean bug is triggered by the infection of the gut symbiont.

### Morphological alteration of the midgut crypts in response to *Caballeronia* colonization

The symbiotic organ of the bean bug consists of hundreds of crypts. Thus, the initial morphological development of the midgut crypts in response to their colonization by the *Caballeronia* gut symbiont was further monitored by confocal laser scanning microscopy (LSM) and transmission electron microscopy (TEM). Our LSM observation revealed that the midgut crypts of aposymbiotic insects were primordial and their epithelial cells were merely aligned without forming an inner lumen (Figure 1A). Similarly, although a small number of *Caballeronia* symbiont cells had already reached the main duct of the symbiotic organ, the midgut crypts of symbiotic insects had not developed at 12 h after symbiont infection (Figure 1A). However, 24 h after symbiont infection, the midgut crypts started to form spacious inner cavities and the *Caballeronia* symbiont cells entered the newly generated crypt lumen (Figure 1A). The M4 region of symbiotic insects continually matured, forming sac-like crypts and *Caballeronia* symbionts proliferated and fully colonized inside of the crypts within 48 h after infection (Figure 1A). In contrast, the midgut crypts of aposymbiotic insects did not modify during the entire second instar nymph stage and only a narrow space of inner lumen was created in the third instar nymph (Figure 1A). TEM imaging also showed that the lumen of the midgut crypts of aposymbiotic insects was constricted, but those of symbiotic insects possessed a vast lumen entirely colonized by the *Caballeronia* symbionts (Figure 1A).

We then measured numerical parameters of the midgut crypts to describe their development in more detail. As observed by LSM, the size of midgut crypts was significantly larger in symbiotic insects than in aposymbiotic insects at both the second and third instar nymph stages (Figure 1B). The total number of the midgut crypts through the symbiotic organ was consistent between aposymbiotic and symbiotic insects, indicating that the *Caballeronia* symbiont induces the development of each midgut crypt but does not trigger formation of additional crypts (Figure 1C). Nevertheless, all the enlarged crypts of the symbiotic organ in symbiotic insects collectively made the entire midgut longer and wider compared to aposymbiotic insects (Supplementary Figures S3A, S3B).

To investigate how *Caballeronia* symbiont triggers midgut development, we estimated indirectly the relative number of nuclei present in the whole symbiotic organ of aposymbiotic and symbiotic insects by quantifying copy numbers of the housekeeping gene *elongation factor 1 alpha* (*ef1ɑ*) by quantitative PCR (qPCR). The total number of nuclei was estimated to be higher in symbiotic insects than in aposymbiotic insects at the second instar nymph stage, and the midgut cells further proliferated at the third instar nymph stage in comparison with the aposymbiotic insects, suggesting that the symbiont-mediated crypt development could be related to the regulation of the cell cycle in the crypt epithelial cells (Figure 1D).

### *Caballeronia* symbiont induces ISC proliferation

**FIGURE 2:**
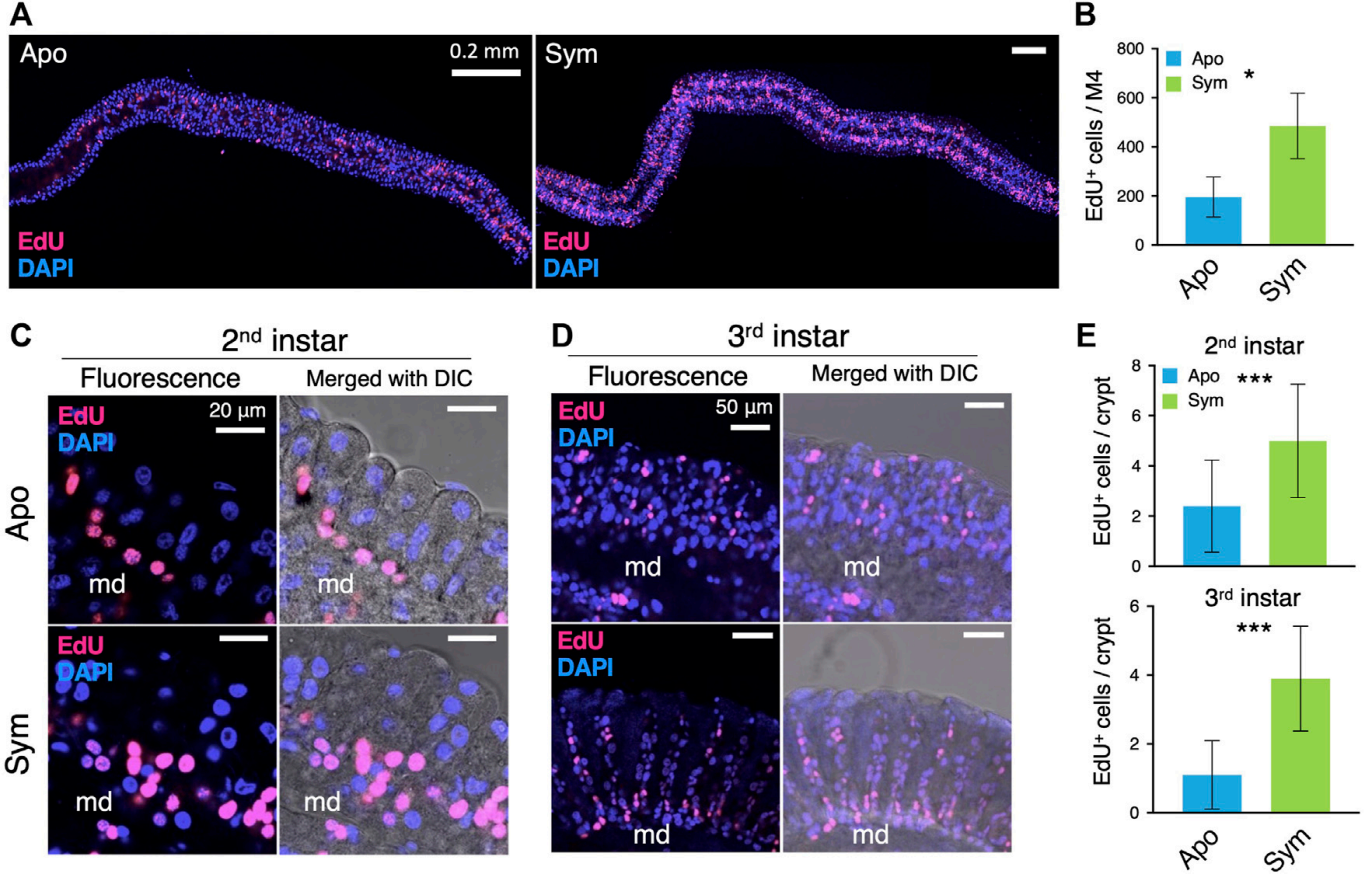
*Caballeronia* symbionts stimulate the proliferation of ISCs. ISCs are visualized by EdU staining (red) and compared between aposymbiotic and symbiotic insects. Blue fluorescence corresponds to host nuclei stained with DAPI. **(A)** Wholemount images of the symbiotic organ. **(B)** The number of EdU-positive (EdU^+^) cells per symbiotic organ (*n* = 3). LSM images of midgut crypts in **(C)** second instar and **(D)** third instar. Merged images of fluorescence and DIC are also shown to visualize crypts and the main duct (md). Note that EdU^+^ cells (i.e. proliferating ISCs) are densely localized at the base part of crypts, which is obvious in symbiotic insects. **(E)** The number of EdU^+^ cells per crypt (*n* = 9) was statistically analyzed by the Mann-Whitney *U* test (*, *p* < 0.05; ***, *p* < 0.001). Apo, aposymbiotic; Sym, symbiotic insects.

The gut epithelium of insects is maintained by the multipotent ISCs. We investigated the number and localization of the ISCs from the symbiotic organ of aposymbiotic and symbiotic insects by using a thymidine analogue, 5-ethynyl-2′-deoxyuridine (EdU), which is incorporated into the newly synthesizing DNA of dividing cells. The LSM images showed that the number of EdU^+^ ISCs along the entire symbiotic organ was lower in aposymbiotic insects than in symbiotic insects (Figure 2A). Indeed, more than twice as much EdU^+^ ISCs were counted in the symbiotic organ of symbiotic insects than in that of aposymbiotic insects, demonstrating that the gut colonization by *Caballeronia* symbiont induces the proliferation of ISCs (Figure 2B). We further identified where the ISCs are localized in the midgut crypts of the bean bug. At the early developmental stage of the second instar nymph, the majority of ISCs were detected at the crypt base in both aposymbiotic and symbiotic insects although the number of EdU^+^ ISCs per crypt was much higher in symbiotic insects compared to aposymbiotic insects (Figures 2C, E). Similarly, the number of EdU^+^ ISCs per crypt was higher in symbiotic insects than in aposymbiotic insects at the third instar nymph, and numerous ISCs were found at the base of the crypts although some Edu^+^ cells were also observed in the middle or at the tip side of the crypts (Figures 2D, E). This result indicates that the gut-colonizing *Caballeronia* symbiont stimulates the proliferation of ISCs.

**FIGURE 3:**
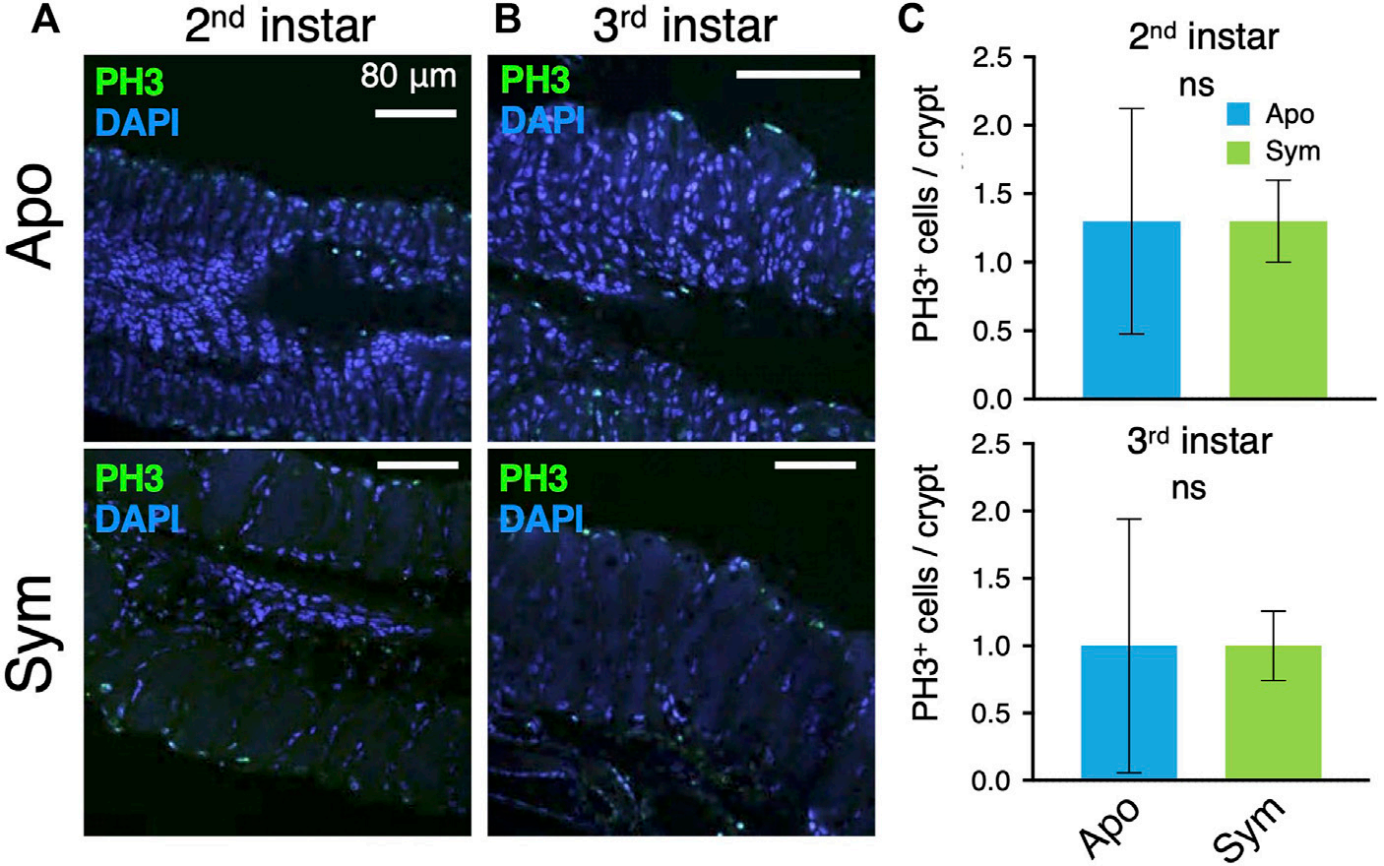
Mitotic cells in symbiotic organs of aposymbiotic and symbiotic insects. Mitotic cells are visualized by immunostaining of PH3. LSM images of **(A)** second instar and **(B)** third instar insects. Green and blue are derived from anti-PH3 and DAPI staining, respectively. **(C)** The number of PH3-positive cells per crypt (*n* = 9). The data was analyzed by the Mann-Whitney *U* test (ns, non-significant). Apo, aposymbiotic; Sym, symbiotic insects.

Next, mitotic cells in the symbiotic organ were observed by immunostaining of the mitotic cell marker Phospho-Histone H3. The number of PH3^+^ cells was basically small (Figures 3A, B) and there were no significant differences between aposymbiotic and symbiotic insects at the second and third instar nymph (Figure 3C). However, the number of nuclei per midgut crypt was significantly higher in the symbiotic insects than aposymbiotic insects (Supplementary Figure S3C), strongly suggesting that *Caballeronia* symbiont stimulates cell division, although mitotic cells were scarcely detected by PH3 antibody in *R. pedestris*.

### *Caballeronia* symbiont suppresses apoptosis in the midgut crypts

**FIGURE 4:**
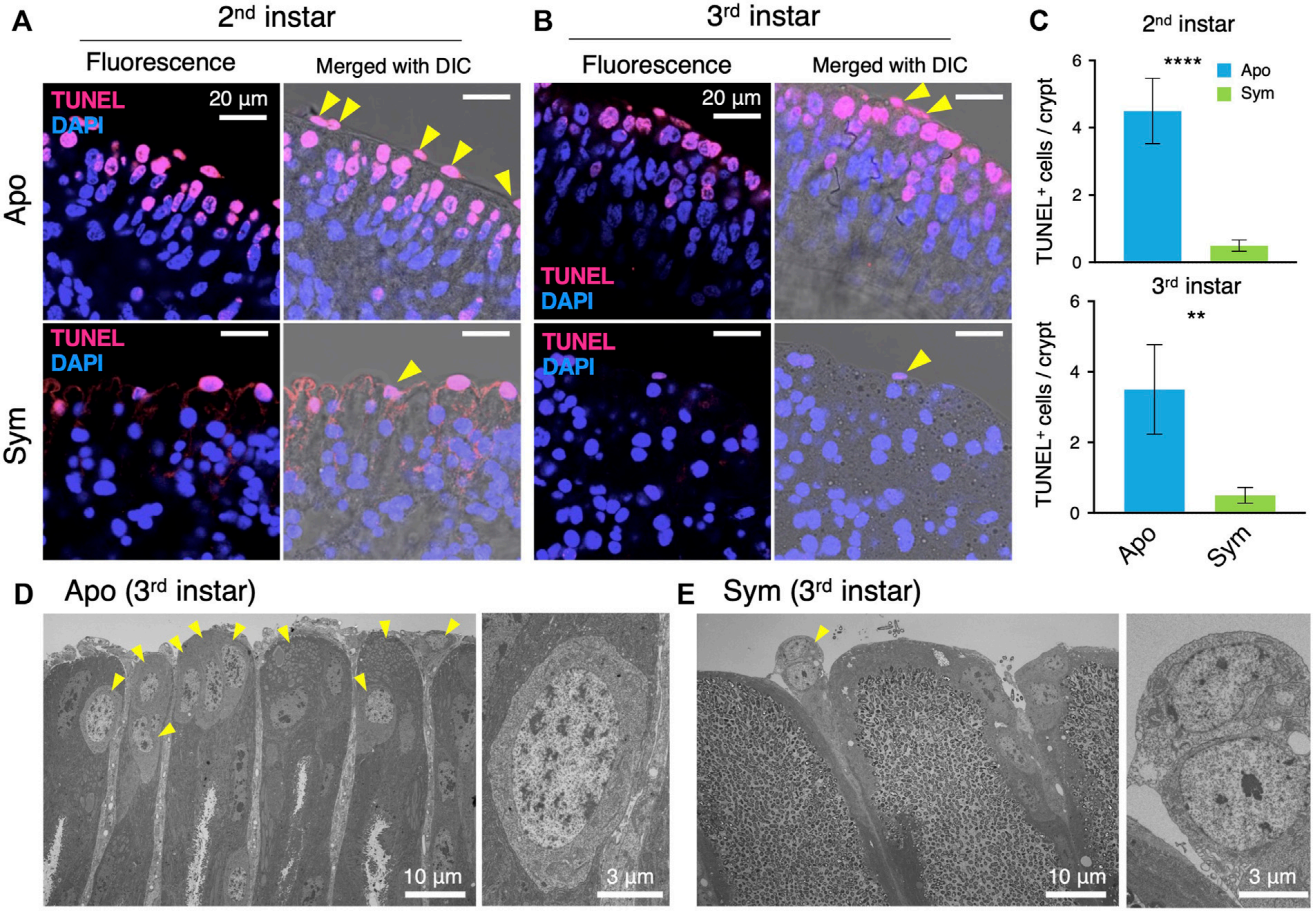
*Caballeronia* symbionts inhibit apoptosis of midgut crypts. Apoptosis is visualized by TUNEL staining (red) and compared between aposymbiotic and symbiotic insects. Blue is DAPI staining of nuclei. LSM images of midgut crypts in **(A)** second instar and **(B)** third instar. Merged images of fluorescence and DIC are also shown to visualize the tip parts of midgut crypts. Note that apoptotic cells are localized at the tip part of crypts and are numerous in aposymbiotic insects. **(C)** The number of apoptotic cells per crypt (*n* = 9). The number of apoptotic cells was statistically analyzed by the Mann-Whitney *U* test (**, *p* < 0.01; ***, *p* < 0.001). TEM images of midgut crypts in **(D)** aposymbiotic and **(E)** symbiotic insects (third instar). Arrowheads indicate cells showing abnormal morphology, which seems to be apoptotic cells. Enlarged images of apoptotic cells are shown on the right. Apo, aposymbiotic; Sym, symbiotic insects.

Since old or excess enterocytes in mammals and insects are removed by apoptosis, we visualized apoptotic cells of the symbiotic organ by TUNEL staining. As opposed to ISCs, the apoptotic cells were detected on the tip side in both aposymbiotic and symbiotic insects (Figures 4A, B). However, there were significantly more TUNEL-derived fluorescence signals of apoptotic cells in aposymbiotic insects than in symbiotic insects (Figure 4C). The apoptotic cells in the symbiotic organ of aposymbiotic insects were even observed in the middle side of the crypts, whereas apoptosis occurred only on the tip side in symbiotic insects (Figures 4A, B). Particularly, the fluorescence signal was strongly detected in cells located in-between the midgut crypts in aposymbiotic insects (Figures 4A, B). The TEM images of the midgut crypts confirmed that numerous cells with abnormal morphology accumulated at the tip side of the crypts in aposymbiotic insects (Figure 4D). The apoptotic cells located between midgut crypts seem to be shed from the symbiotic organ into the hemolymph. However, in symbiotic insects, apoptotic cells in the midgut crypts were barely observed by TEM and only a few cells were released from the crypts (Figure 4E).

## Discussion

In the stinkbug-*Caballeronia* symbioses, midgut crypts differentiate markedly and mature along with the symbiont colonization (Kikuchi et al., 2007; 2011b). Although the morphological change has been well-characterized, the detailed cellular process and cytological features of crypt morphogenesis remained unclear. By visualizing cell proliferation, mitosis, and apoptosis of midgut crypts by molecular markers in the *Riptortus*-*Caballeronia* model system, we demonstrated in this study that: 1) ICSs in the symbiotic organ were located and proliferated at the crypt base; 2) differentiated enterocytes migrated upward along the crypt axis as the midgut developed, apoptotsis of aged enterocytes occurred at the crypt tip, and apoptotic cells eventually seemed to be shed from the crypts into the hemolymph; 3) the proliferation rate of the ISCs was low in aposymbiotic insects, and conversely, the apoptotic rate was high, resulting in undeveloped, short crypts; on the other hand, 4) gut-colonizing *C. insecticola* promoted the proliferation of the ISCs at the crypt base and simultaneously inhibited cell apoptosis at the tip of the crypts, resulting in enlarged crypts. These results clearly demonstrated that the *Caballeronia* symbiont stimulates and coordinates the development of midgut crypts *via* regulating the epithelial cell cycles, thereby facilitating stable and ample symbiont colonization into the generated spacious crypts of the bean bug host. This crypt developmental response that we describe here adds up to two other symbiosis-induced developmental responses in the midgut that we identified before. The first one is the closure of the constricted region a few hours after the passage of the initial bacterial colonizers, preventing subsequent infections of the symbiotic organ by other bacteria (Kikuchi et al., 2020). The second one consists in the development of an extensive network of tracheae that envelope the symbiotic organ in response to its colonization, enabling the oxygenation of the symbiont population in the crypts (Jang et al., 2021).

The squid-*Vibrio* symbiosis is one of the most well-investigated model systems for animal-microbe symbiosis, wherein a symbiont-stimulated drastic morphological change of a bioluminescent symbiotic organ has been described (McFall-Ngai and Ruby, 1991; Montgomery and McFall-Ngai, 1994; McFall-Ngai, 2014). In the squid host, symbiont infection induces apoptosis of the anterior appendage of the luminescent organ, which is not essential for symbiont accommodation but induces the maturation of the rest of the symbiotic organ (Foster and McFall-Ngai, 1998; Foster et al., 2000). The phenomenon we found here in the bean bug is comparable but at the same time quite distinct: apoptotic cells are abundant in the tip part of underdeveloped crypts in aposymbiotic insects, while the apoptosis is strongly suppressed once *Caballeronia* symbiont colonizes. In contrast to the squid-*Vibrio* symbiosis, the suppression of apoptosis plausibly plays a pivotal role in the enlargement and maturation of the symbiotic organ in the bean bug host. Remarkably, many pathogenic bacteria in fact inhibit apoptosis in the course of infection in such a way that they can stably colonize and proliferate inside host cells (Faherty and Maurelli, 2008). Cell apoptosis commonly occurs by activation of the caspase family including caspase-3 and pathogenic bacteria inhibit the caspase-activation pathway by various molecular inhibitors (Faherty and Maurelli, 2008). A comparison with the survival strategy of these pathogenic bacteria could give a hint to clarify the mechanism in the interaction of the *Caballorinia* symbionts with the midgut epithelia. While suppression of the apoptosis in midgut crypts may strengthen the bond between *Riptortus* and *Caballeronia*, the high apoptotic rate of aposymbiotic bugs might imply that excessive crypt cells are lysed and recruited for nutritional compensation of the absence of symbiotic bacteria whose role is to supplement the host diet (Ohbayashi et al., 2019a). Such phenotypic plasticity in the stinkbug-*Caballeronia* symbioses may also contribute to the flexibility in partner selection in the face of the myriad of environmental bacteria that potentially can infect the crypt region of the midgut and allow to adjust crypt morphology until the host nymphs find the best partner (Itoh et al., 2019).

Besides the suppression of apoptosis in the tip part of crypts, the gut-colonizing *Caballeronia* symbiont simultaneously induces cell divisions of ISCs at the crypt base, revealed mostly by the enhanced signal from the EdU marker of DNA replication. PH3 on the other hand is a well-known marker of mitosis (Hirota et al., 2005; Lin et al., 2008; Buchon et al., 2009) and immunostaining of PH3 has been widely used in numerous insect species including *Drosophila*, termites and planthoppers to reveal mitotic cells (Buchon et al., 2009; Sousa et al., 2019; Guo et al., 2020). In this study, we performed the immunostaining of PH3 to reveal the localization of mitotic cells in midgut crypts. Only a few PH3^+^ cells were distributed both in the base and tip parts of the symbiotic organ because histone H3 Ser-10 phosphorylation occurs during cell apoptosis as well as mitosis (Waring et al., 1997; Füllgrabe et al., 2010), but there was no significant difference between symbiotic and aposymbiotic insects. This result, which is distinct from the EdU-staining, may indicate that the cell division is a quick process in both aposymbiotic and symbiotic insects, and consequently mitotic cells between the G2 and M phase may be scarce in the cell population of midgut crypts. Likewise, PH3^+^ cells are barely observed in the gut of other insects (Bonelli et al., 2019; Janeh et al., 2019; Tian et al., 2022) or even not observed (Huang et al., 2016). In this context, it should be noted that the number of cells per midgut crypt was significantly increased in the symbiotic insects compared to the aposymbiotic insects, indicating that *Caballeronia* symbiont promotes cell proliferation in the midgut crypts.

**FIGURE 5:**
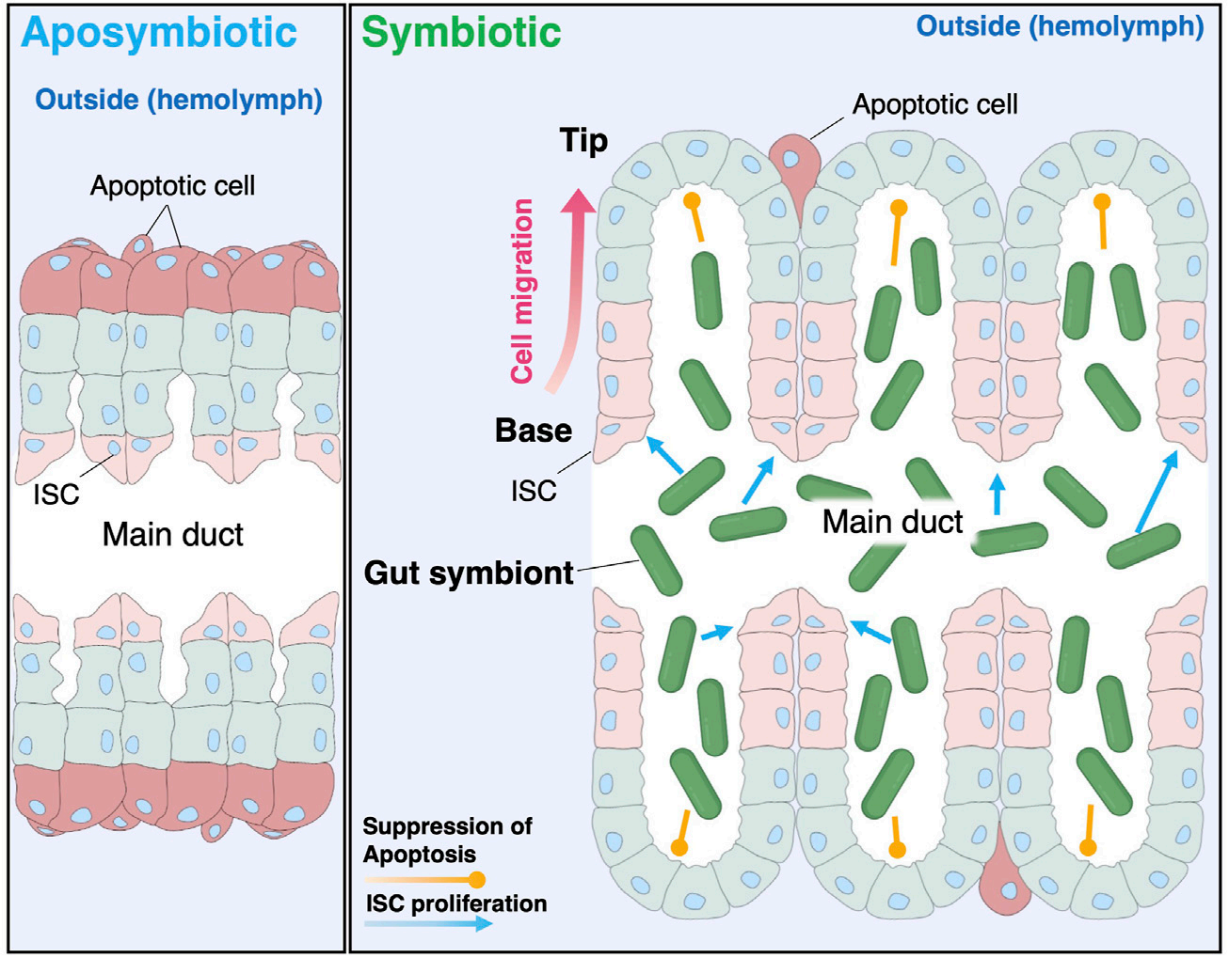
Schematic illustration of the symbiont-mediated crypt morphogenesis in the bean bug. *C. insecticola* stimulates the proliferation of ISCs localized at the crypt base and simultaneously inhibits apoptosis at the crypt tip, resulting in the dramatic alteration of the crypt morphogenesis in the bean bug *R. pedestris*.

In several animal models, gut microbiota contributes to ISC proliferation and gut homeostasis. In the case of the fruit fly *D. melanogaster*, when the digestive tract is damaged by pathogenic bacteria, ISC proliferation is activated *via* the JAK-STAT pathway, leading to the repair of the intestinal epithelium (Apidianakis et al., 2009; Buchon et al., 2009; Jiang et al., 2009; Bonfini et al., 2016). Interestingly, commensal gut bacteria also activate ISC proliferation through the immune pathways (Buchon et al., 2009; Bonfini et al., 2016). However, distinct to the here-described bacterial-induced crypt differentiation in *R. pedestris* where the balance between ISC proliferation and apoptosis of enterocytes changes in favor of more cell generation and thus enlargement of the crypts, in the *D. melanogaster* gut, cell proliferation and apoptosis are maintained in a homeostatic compensatory mechanism preserving gut morphology (Ohlstein and Spradling, 2006; Apidianakis et al., 2009; Loudhaief et al., 2017). Another remarkable distinction is that while the midgut in *D. melanogaster* loses cells by delamination of apoptotic cells into the gut lumen (Ohstein and Spradling, 2006; Loudhaief et al., 2017), the apoptotic cells in the *R. pedestris* crypts seems to be shed on the opposite side, into the hemolymph. It should be clarified in further studies how this shedding of apoptotic cells to hemocoel could occur in the presence of basal lamina in the midgut crypts. In mammals, gut commensals are also involved in stem cell proliferation and tissue regeneration (Nigro et al., 2014; Nigro and Sansonetti, 2015), and promote the development of the vasculature system (Stappenbeck et al., 2002; Hooper, 2004). In the mammalian gut, ISCs are localized at the bottom of intestinal crypts, and progenitor cells migrate to the tip of villi and are shed from the tip into the luminal region (Gehart and Clevers, 2019). Although the direction of migration and the luminal cavity is reversed in the bean bug crypts, analogous cell proliferation and shedding patterns seem to govern the cell turnover in the midgut crypts (Figure 5).

Our observations of an inverted localization of the ISCs at the base rather than the tip of the crypts, the localization of the ISCs facing the lumen of the crypts rather than the hemolymph and the shedding of the apoptotic cells in the hemolymph rather than the crypt lumen suggest to us that the M4 crypt region of the *R. pedestris* gut has in fact an inverted orientation compared to the normal, digestive insect midgut. Such an inversion could be a requirement for the distinct function of this symbiotic region of the midgut. Indeed, while in the digestive regions of the insect midgut, the epithelial cells absorb nutrients from the lumen and secrete them into the hemolymph (Miguel-Aliaga et al., 2018), in the symbiotic midgut region, no food is passing and digested (Ohbayashi et al., 2015) but on the contrary, the flux of nutrients is reversed and is directed from the hemolymph towards the crypt lumen in order to feed the symbiotic bacteria with host-provided nutrients (Ohbayashi et al., 2019b). An implication of this “inverted gut hypothesis” is that the apical-basal polarity of the gut epithelial cells is reversed in the crypt epithelia: the apical membrane (facing the midgut lumen) and basolateral membrane (facing the hemolymph) have distinct transport systems assuring nutrient flux from the lumen towards the hemolymph in the digestive midgut and we predict that this orientation is inverted in the symbiotic midgut region, resulting in transport of nutrients from the hemolymph towards the lumen of the crypts. This hypothesis could be tested in the future by comparing in the M4 epithelia and the epithelia of the digestive regions of the midgut, the subcellular distribution of available polarity markers (Chen et al., 2018).

Midgut crypts are commonly found in phytophagous stinkbug species belonging to the infraorder Pentatomomorpha, although there is a certain morphological variation (Kikuchi et al., 2008; 2011a). Members of the superfamily Pentatomidae develop midgut crypts in four rows, in which vertically-transmitted gammaproteobacterial symbionts colonize (Kikuchi et al., 2012; Karamipour et al., 2016; Oishi et al., 2019; Cossolin et al., 2020). Members of the superfamily Coreoidae harbor *Caballeronia* symbionts in midgut crypts organized in two rows (Ohbayashi et al., 2019b; Ishigami et al., 2021; Ohbayashi et al., 2022), as shown in the bean bug. Members of the superfamily Lygaeoidea carry *Caballeronia* symbionts in elongated tubular-form midgut crypts (Kikuchi et al., 2011a; Ishigami et al., 2022). Despite this morphological diversity, considering that the developmental site of crypts is common to the posterior region of the midgut (i.e. M4 region), it is plausible that a similar symbiont-mediated induction of crypt development occurs in the diverse stinkbug species. Although it is still unclear how stinkbug hosts recognize their symbiotic bacteria in the gut symbiotic organ and what molecule alters the crypt’s cell cycle, given the genetic diversity of symbiotic bacteria, including gamma- and beta-proteobacteria, the morphological alteration may be triggered by a simple molecule common to these bacteria, such as lipopolysaccharide, peptidoglycan, and its derivatives. Such symbiont-derived molecules could also signal the closure of the constructed region (Kikuchi et al., 2020). Indeed, peptidoglycan and lipopolysaccharide of symbiotic bacteria contribute to the morphological alteration of the symbiotic organ in squid-*Vibrio* symbiosis (Foster et al., 2000; Troll et al., 2009; Altura et al., 2011). It is remarkable that the putative symbiont signals trigger distinct responses in the crypt epithelia depending on the location of the cells: cell proliferation at the base of the crypts or apoptosis inhibition at the tip of the crypts. This suggests that the different cell types in these two locations activate distinct pathways in response to the bacterial signals (Figure 5). It remains to be seen if these cell types respond to the same bacterial signal or if different signals are involved. In addition to such MAMPs (microbe-associated molecular patterns), symbiont metabolism could also affect the morphogenesis of host insects. For instance, our previous study demonstrated that the *Caballeronia* symbiont consumes luminal oxygen and the induced hypoxic condition activates the tracheal development in the symbiotic organ (Jang et al., 2021). Determination of the trigger that induces the crypt morphogenesis, in conjunction with identification of the recognition mechanism in the host would be of interest and important to understand how insects, which have primarily evolved innate immunity to counter pathogens, can select out a limited number of beneficial symbiotic bacteria from the enormously diverse environmental bacteria.

## Data Availability

The original contributions presented in the study are included in the article/Supplementary Material, further inquiries can be directed to the corresponding authors.
