# Supplementary material for: Symbiont coordinates stem cell proliferation, apoptosis, and morphogenesis of gut symbiotic organ in the stinkbug-Caballeronia symbiosis
Source: Front Physiol. 2023 Jan 4;13:1071987. doi: 10.3389/fphys.2022.1071987 (PMC9846216; doi:10.3389/fphys.2022.1071987)
Supplement: Supplementary file 1 [file DataSheet1.pdf]

Fig. S1

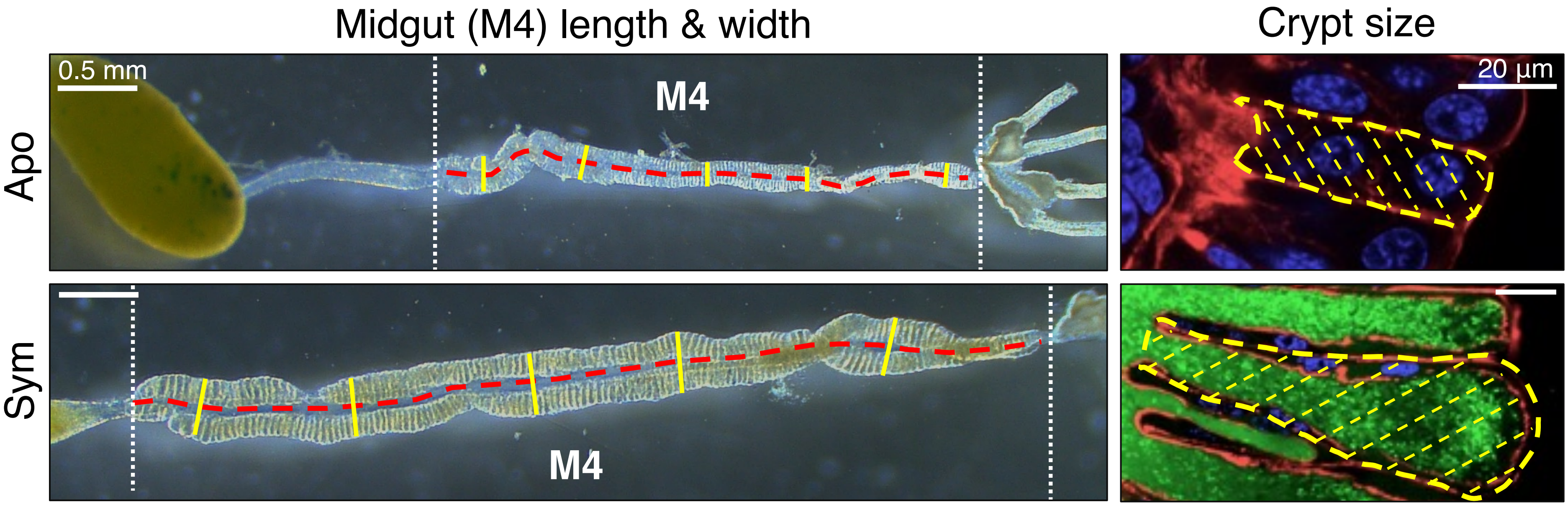

Supplementary figure S1. Methods for measurement of crypt size.
Based on microscopy images, crypt width and length were calculated using line selection tools of ImageJ software. In case of crypt width, the widths of 10 random points of the M4 were measured and the average value was used. The total area of each crypt was measured using area selection tools of ImageJ software. The results are shown in Fig. 1B and Fig. S3.

Fig. S2

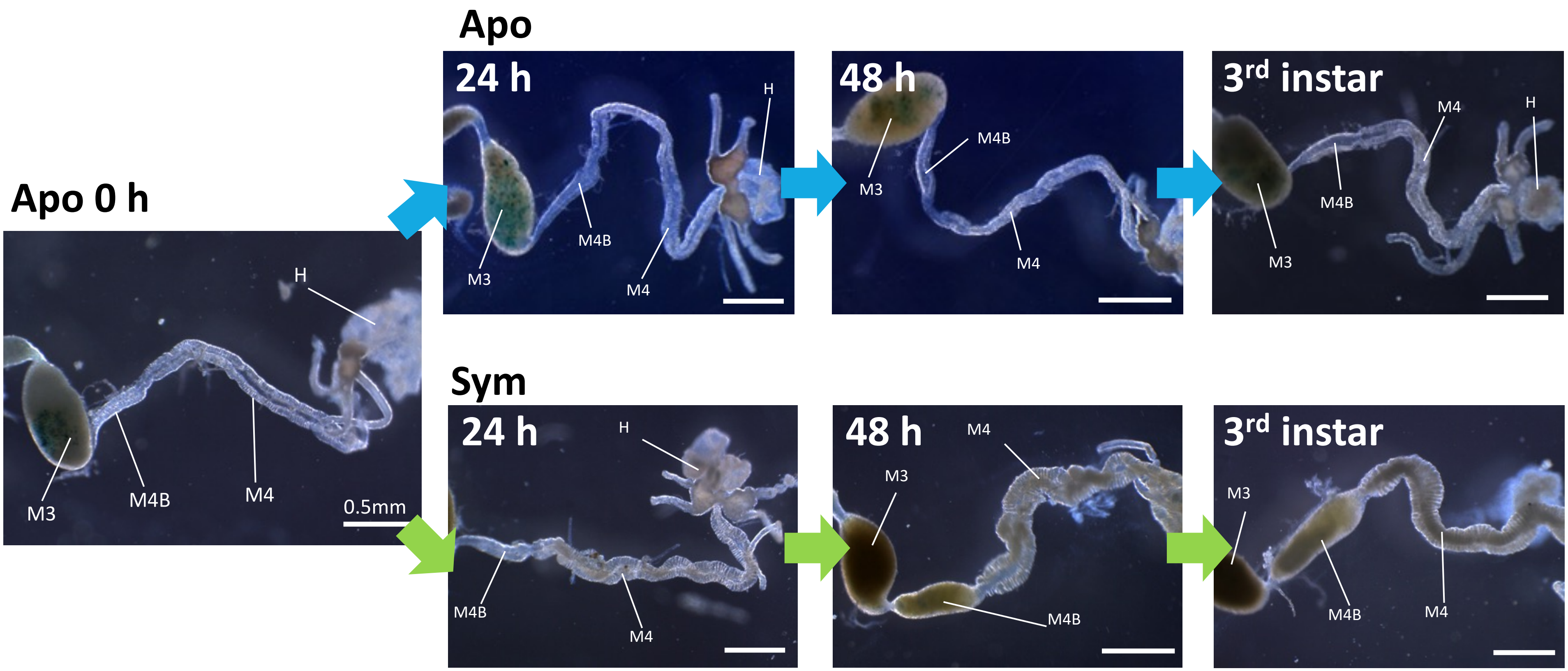

Supplementary figure S2. Morphological alteration induced by *Caballeronia* symbionts in the crypt-bearing midgut region (M4).

While M4 does not strongly change in aposymbiotic insects even in the third instar stage, its morphology dramatically changes in symbiotic insects. In addition to M4, M4 bulb (M4B), which does not develop crypts but digests excess symbiont cells flowing back from M4, also becomes enlarged.

Fig. S3

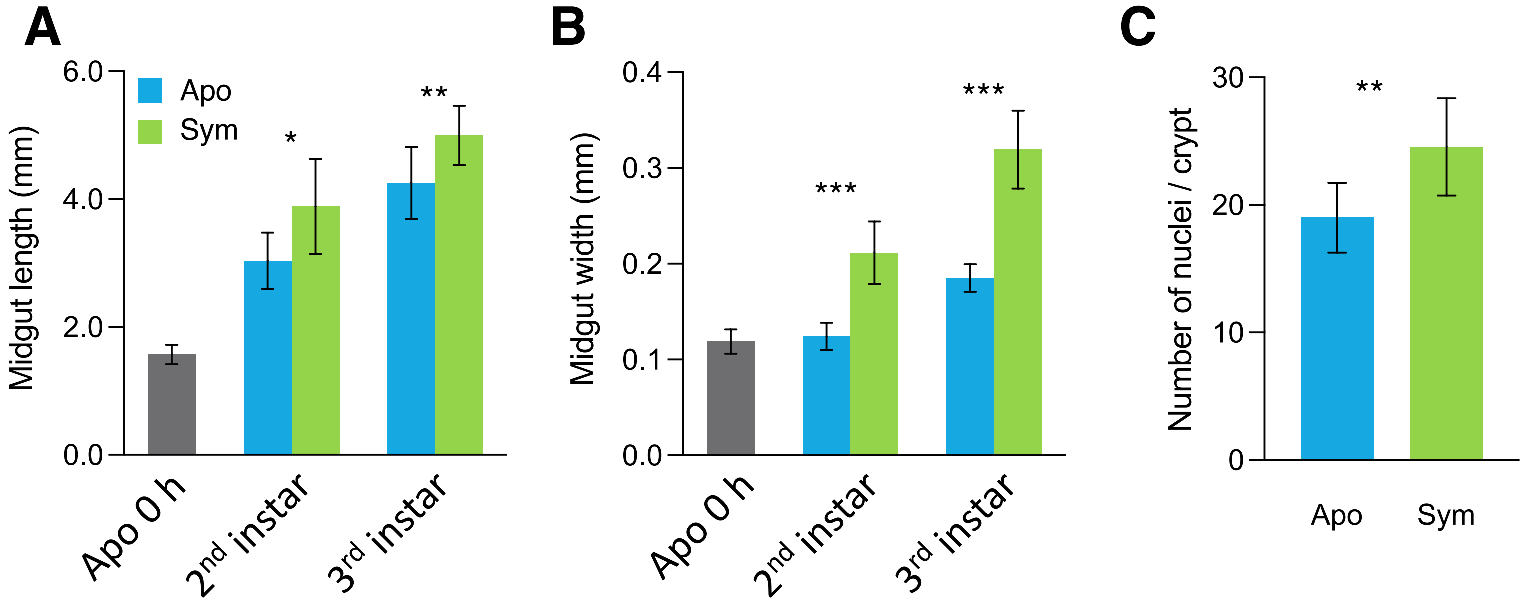

Supplementary figure S3. *Caballeronia* symbionts alter M4 length and width. (A) M4 length and (B) width (n = 10, respectively) gradually increase during development and are strikingly enhanced by symbiont colonization. (C) The number of cells per midgut crypts (n = 9) was measured from aposymbiotic and symbiotic insects. The parameters were statistically analyzed by the Mann-Whitney *U* test (*, $p < 0.05$; **, $p < 0.01$; ***, $p < 0.001$).
